# Prediction of Seven Artificial Intelligence-Based Intraocular Lens Power Calculation Formulas in Medium-Long Caucasian Eyes

**DOI:** 10.3390/life15010045

**Published:** 2025-01-01

**Authors:** Wiktor Stopyra, Oleksiy Voytsekhivskyy, Andrzej Grzybowski

**Affiliations:** 1 MW-Med Eye Centre, 31-416 Krakow, Poland; 2 Department of Medicine, University of Applied Sciences, 34-400 Nowy Targ, Poland; 3 Kyiv Clinical Ophthalmology Hospital Eye Microsurgery Center, 03680 Kyiv, Ukraine; iolcalculation@gmail.com; 4 Institute for Research in Ophthalmology, Foundation for Ophthalmology Development, 61-553 Poznan, Poland; ae.grzybowski@gmail.com; 5 Department of Ophthalmology, University of Warmia and Mazury, 10-720 Olsztyn, Poland

**Keywords:** phacoemulsification, medium-long eyes, intraocular lenses, artificial intelligence, Hill-RBF formula

## Abstract

**Purpose:** To compare the accuracy of seven artificial intelligence (AI)-based intraocular lens (IOL) power calculation formulas in medium-long Caucasian eyes regarding the root-mean-square absolute error (RMSAE), the median absolute error (MedAE) and the percentage of eyes with a prediction error (PE) within ±0.5 D. **Methods:** Data on Caucasian patients who underwent uneventful phacoemulsification between May 2018 and September 2023 in MW-Med Eye Center, Krakow, Poland and Kyiv Clinical Ophthalmology Hospital Eye Microsurgery Center, Kyiv, Ukraine were reviewed. Inclusion criteria, i.e., complete biometric and refractive data, were applied. Exclusion criteria were as follows: intraoperative or postoperative complications, previous eye surgery or corneal diseases, postoperative BCVA less than 0.8, and corneal astigmatism greater than 2.0 D. Prior to phacoemulsification, IOL power was computed using SRK/T, Holladay1, Haigis, Holladay 2, and Hoffer Q. The refraction was measured three months after cataract surgery. Post-surgery intraocular lens calculations for Hill-RBF 3.0, Kane, PEARL-DGS, Ladas Super Formula AI (LSF AI), Hoffer QST, Karmona, and Nallasamy were performed. RMSAE, MedAE, and the percentage of eyes with a PE within ±0.25 D, ±0.50 D, ±0.75 D, and ±1.00 were counted. **Results:** Two hundred fourteen eyes with axial lengths ranging from 24.50 mm to 25.97 mm were tested. The Hill-RBF 3.0 formula yielded the lowest RMSAE (0.368), just before Pearl-DGS (0.374) and Hoffer QST (0.378). The lowest MedAE was achieved by Hill-RBF 3.0 (0.200), the second-lowest by LSF AI (0.210), and the third-lowest by Kane (0.228). The highest percentage of eyes with a PE within ±0.50 D was obtained by Hill-RBF 3.0, LSF AI, and Pearl-DGS (86.45%, 85.51%, and 85.05%, respectively). **Conclusions:** The Hill-RBF 3.0 formula provided highly accurate outcomes in medium-long eyes. All studied AI-based formulas yielded good results in IOL power calculation.

## 1. Introduction

Patients’ expectations of correct sight after cataract surgery continue to rise, so an accurate intraocular lens (IOL) power calculation is a very important aspect of phacoemulsification [1]. However, according to the European Registry of Quality Outcomes in Cataract and Refractive Surgery, the percentage of patients with a prediction error (PE) within ±0.5 D after cataract surgery is only 73.7% [2]. This may be due to errors in biometric measurements such as anterior chamber depth (ACD), axial length (AL), and keratometry (K), which can cause 42%, 36%, and 22% of mistakes, respectively [3]. But most of all, it relies on effective lens position (ELP) estimation resulting from choosing a relevant IOL power calculation formula [4,5].

We currently use a lot of IOL power calculation formulas, including artificial intelligence (AI)-based ones [6,7,8]. However, despite so many formulas, there is still no gold standard regarding the choice of the appropriate formula [9,10,11,12,13,14].

The FullMonte method, the first AI-based IOL power calculation formula, was introduced by Gerald Clarke [15]. As a hybrid, in addition to optical components, it used a Monte Carlo Markov Chain simulator to improve its accuracy [16]. To date, many more formulas using AI have been developed [17,18,19,20,21,22,23,24]. We can divide them into methods applying AI only and hybrids, i.e., formulas that are based on an optical approach or ray tracing and that use AI to refine it. In addition, there are universal algorithms utilizing AI to improve the trueness of existing formulas. AI applying in IOL power calculation is summarized in Table 1. There are many studies on AI-based IOL power calculation formulas; however, they compare the accuracy of four [20], five [25,26], or, at most, six such formulas [27].

Since myopic eyeballs can range in length, there is no consensus regarding their lower limit [7,8,12,13,14]. The following ALs are considered, i.e., 24.0 mm [28], 24.5 mm [29], 25.0 mm [30,31], 25.5 mm [32,33], or even 26.0 mm [20,34,35]. Therefore, the range of AL between 24.5 mm and 25.99 mm is referred to as medium-long eyes [16,25,36,37]. So far, medium-long eyes have been examined rarely in studies on the trueness of IOL power calculation formulas.

The accuracy of an IOL power calculation formula can be assessed by various parameters. Most studies use the mean absolute error (MAE) and the percentage of eyes with a PE within ±0.50 D [16,29,32,34,35,37]. Hoffer et al. consider the median absolute error (MedAE) as the main outcome measure [38]. In turn, Holladay et al. propose the root-mean-square absolute error (RMSAE), which is benchmarked between formulas utilizing the bootstrap-t method with Holm sequential correction [39,40]. It is an option to SD for describing the distribution of PEs for subgroups such as long eyes, short eyes, and eyes with keratoconus or after corneal refractive surgery, i.e., with non-zero predicted errors [40].

This study was designed to compare seven AI-based IOL power calculation formulas for medium-long eyes (24.50 mm ≤ AL ≤ 25.99 mm) using RMSAE, MedAE, and the percentage of eyes with a PE within ±0.25 D, ±0.50 D, ±0.75 D, and ±1.00 D as the main outcome measures. To our knowledge, this is the first study applying such a methodology comparing as many as seven AI-based formulas. Moreover, the study refers to medium-long eyes, which is a rarity, and there are only a few peer-reviewed articles on this topic but with a much smaller sample size [16,25].

## 2. Material and Methods

We retrospectively analyzed the data of patients with ALs between 24.50 mm and 25.97 mm who underwent uneventful sutureless cataract surgery with a 2.4 mm clear corneal incision and had implanted a monofocal IOL between May 2018 and September 2023 in MW-Med Eye Center, Krakow, Poland and Kyiv Clinical Ophthalmology Hospital Eye Microsurgery Center, Kyiv, Ukraine. The following exclusion criteria were applied, i.e., intraoperative or postoperative complications, postoperative BCVA less than 0.8, corneal astigmatism greater than 2.0 D, and previous eye surgery or corneal diseases.

The study was conducted adhering to the tenets of the Declaration of Helsinki and was approved by the Institutional Review Board of the Foundation for the Advancement of Ophthalmology “Ophthalmology 21” (7/2024). Prior to phacoemulsification, each patient signed informed consent for surgery.

For each patient, preoperative biometry data such as AL, ACD, K, white to white (WTW) as corneal diameter and lens thickness (LT) were obtained utilizing a Zeiss IOLMaster 700 (Carl Zeiss Meditec AG, Jena, Germany), while central corneal thickness was achieved using an auto kerato-refractonometer TRK-2P (Topcon Corporation, Tokyo, Japan). Before phacoemulsification, IOL power was computed applying the following formulas: SRK/T, Hoffer Q, Holladay 1, Haigis, Holladay 2, and often Barrett Universal II. We randomly selected the power of the implanted IOL from the outcomes of SRK/T, Holladay 2, or Barrett Universal II. Two surgeons performed all cataract surgeries. They implanted only acrylic foldable IOLs (Acrysof IQ SN60WF—Alcon Laboratories, Fort Worth, TX, USA). Three months after cataract surgery, postoperative refraction was measured at 6 m, as recommended by Simpson and Charman [41]. Both pre-surgery and post-surgery measurement data were taken by experienced medical staff. Then, we computed post-surgery IOL power using seven AI-based formulas such as Hill-RBF 3.0, Hoffer QST, Kane, Karmona, LSF AI, PEARL-DGS, and Nallasamy. We excluded FullMonte (inactive online calculator), Zhu-Lu (designed for eyes with AL > 26.0 mm), and Zeiss AI (unavailable online calculator) [22]. We did not zero mean the PE since the AcrySof IQ SN60WF IOL has optimized constants derived from a huge data group, so we utilized these constants from the ULIB website. The A-constant of 119.00 and keratometric index of 1.3375 were used for all methods. We calculated implant power for AI-based formulas using the corresponding web resources (https://www.rbfcalculator.com/ accessed on 11 December 2024, https://www.iolformula.com/ accessed on 11 December 2024, https://www.iolsolver.com/regular, accessed on 11 December 2024, https://www.iolcalc.com/, accessed on 11 December 2024, https:/hofferqst.com/, accessed on 11 December 2024, https://www.lenscalc.com/ accessed on 11 December 2024 and https://karmona-iol.com/ accessed on 11 December 2024).

PE was defined as the difference between the actual postoperative refraction (spherical power plus half the cylindrical power) and the predicted refraction. Absolute error (AE) was determined as an absolute value of PE. Using AE, the percentage of patients with a PE within ±0.25 D, ±0.50 D, ±0.75 D, and ±1.00 D was calculated.

We analyzed data in IBM SPSS Statistics for Windows, Version 22.0 (IBM Corp., Armonk, NY, USA) and R Project 4.3.0 for Statistical Computing (https://www.r-project.org/). We evaluated the normality of PE distribution using the Kolmogorov–Smirnov test. We applied the RMSAE (an alternative to SD for describing the distribution of PEs) and the mean of PE as primary outcomes. We utilized the bootstrap-t method with Holm sequential correction to compare the RMSAE between formulas. Additionally, MedAE was considered. A *p*-value less than 0.05 was considered statistically significant unless the Bonferroni correction was applied. We compared the percentage of eyes with a PE within ±0.50 D using a nonparametric Cochran Q test with the McNemar post hoc test. We calculated the minimum sample size of 138 eyes to achieve a confidence level of 95% (PS program, Version 3.0.12; Dupont WD, 2012).

## 3. Results

Two hundred fourteen eyes (90 male and 124 female) were included in the study. The AL of the examined eyes ranged from 24.50 mm to 25.97 mm. Demographic data of the comprised patients are summarized in Table 2.

Most formulas achieved somewhat positive PEs, except for Nallasamy (−0.021). It should be emphasized that the positive PE outcomes were very close to zero, except Karmona (0.185). Hill-RBF 3.0 and LSF AI, on the other hand, obtained a PE close to zero (0.008).

**Figure 1:**
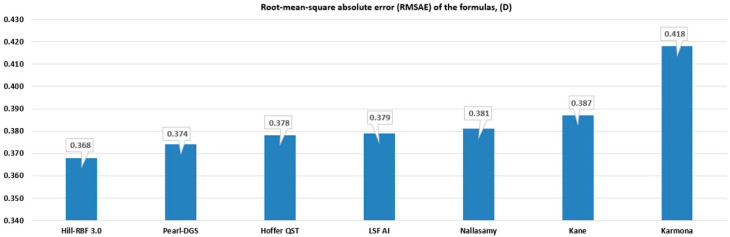
Root-mean-square absolute error (RMSAE) of the studied formulas.

Out of seven evaluated formulas, Hill-RBF 3.0 obtained the lowest RMSAE (0.368), followed by PEARL-DGS (0.374) and Hoffer QST (0.378). In turn, Karmona yielded the highest RMSAE (0.418). The RMSAE outcomes of each formula are listed in Figure 1. The RMSAE statistical comparison was realized utilizing the Bootstrap-t method with the Holm correction. A *p*-value less than 0.05 was statistically significant and was found only for the Hill-RBF 3.0 vs. Karmona (*p* = 0.021). Detailed outcomes are presented in Table 3.

**Figure 2:**
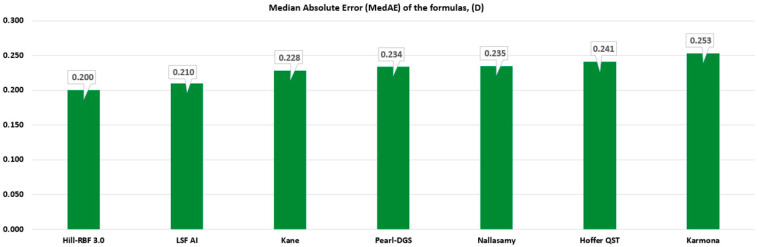
Median absolute error (MedAE) of the studied formulas.

In terms of the MedAE, the best outcomes were achieved by Hill-RBF 3.0 (0.200), LSF AI (0.210), and Kane (0.228), and the worst by Karmona (0.253), as shown in Figure 2.

**Figure 3:**
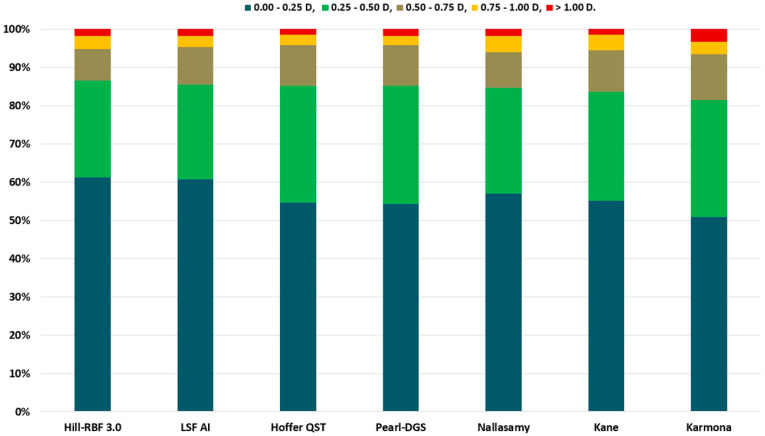
Percentage of eyes with prediction error (PE) within ±0.25 D, ±0.50 D, ±0.75 D, and ±1.00 D.

The highest percentage of patients with PEs within ±0.50 D was found for Hill-RBF 3.0 (86.45%), followed by LSF AI (85.51%) and PEARL-DGS (85.05%). Karmona, on the other hand, gained the smallest percentage of patients with PEs within ±0.50 D (81.31%), as shown in Figure 3. However, multiple comparisons of the formulas according to the Cochran Q test with the McNemar post hoc paired test did not find statistical significance (*p* > 0.271).

In turn, Kane and Hoffer QST obtained the highest percentage of patients with a PE within ±1.00 D (98.60%). The optimized constants, PE, RMSAE, MAE, MedAE, SD, and mean absolute deviation (MAD), as well as the percentage of patients with a PE within ±0.25 D, ±0.50 D, ±0.75 D, and ±1.00 D and the outcomes of all examined formulas are listed in Table 4.

## 4. Discussion

This study demonstrated that out of seven AI-based IOL power calculation formulas, the Hill-RBF formula achieved the lowest RMSAE (0.368), the lowest MedAE (0.200), and the highest percentage of patients with a PE within ±0.50 D (86.45%). This is consistent with Nemeth et al. 2022 study [42]. They concluded that Hill-RBF is more accurate than Kane and PEARL-DGS. They achieved MAE, MedAE, and a percentage of patients within a PE ± 0.50 D almost the same as us (0.30, 0.18, and 80.70, respectively), while our results were 0.27, 0.20, and 86.45, respectively. Slightly worse outcomes of their basic parameters, such as MAE and the percentage of patients within a PE ± 0.50 D, are due to the fact that they used Hill-RBF 2.0, and we utilized Hill-RBF 3.0. It is proved that Hill-RBF 3.0 is more accurate than Hill-RBF 2.0 [43]. The study of Nemeth et al. has some limitations, i.e., a comparison of only three AI-based formulas, a small sample size (153 eyes), and an application of the procedure for subjective refraction, which is not perfectly repeatable and not free of bias (known as invariant refraction assumption) [38].

The Hill-RBF formula was launched in 2016 by Warren E. Hill, MD, as the first AI-based method utilizing radial basis functions. This innovative approach is entirely data-driven, leveraging pattern recognition techniques developed in Matlab and advanced data interpolation methodologies. It uses a large dataset (more than 12,000 eyes in version 2.0 and over 30,000 eyes in version 3.0). Initially, Hill-RBF refused to provide a refractive prediction if it was likely to be inaccurate, and early versions were restricted to Plano target refraction. Currently, it provides the prediction always, just appending a warning sign next to it when the outcome is doubtful. As a pure data-driven formula, it is free of the restrictions and benefits of a lens-position evaluation. This formula has an online calculator. Key inputs for the Hill-RBF 3.0 formula include AL, K, ACD, LT, WTW, CCT, and gender [44].

The first studies on the accuracy of IOL power in medium-long eyes concerned third-generation formulas. Hoffer et al. involved 52 such eyes, obtaining the lowest MAE for SRK/T ahead of Holladay 1 and Hoffer Q (0.345, 0.368, and 0.465, respectively) [45]. Narváez et al., comprising 317 such eyes, found no significant difference in the accuracy of formulas, achieving this same MAE for SRK/T and Holladay 1 (0.49) [46]. However, at the time, they achieved biometric data utilizing an ultrasound immersion technique, which is less precise than swept optical coherence tomography accessible at the IOL-Master device [47]. Aristodemou et al. included 712 such eyes, showing only a trend toward lower MAEs for Holladay 1, with statistical differences in both IOL subgroups, i.e., Akreos Fit and Sopfort AO (Bausch & Lomb, Rochester, NY, USA) [48].

Then, fourth-generation formulas, such as Holladay 2, Haigis, or Barrett Universal II, were considered in studies on IOL power calculation in medium-long eyes. In addition, biometric data were achieved by applying an IOL-Master, which raised the trueness of the biometric measurements. Kane et al. enrolled 372 such eyes, proving the superiority of Barrett Universal II. In their study, Barrett Universal II gained the lowest MAE, followed by T2 and Holladay 1 (0.338, 0385, and 0385, respectively) [49]. The Voytsekhivskyy survey, including 70 such eyes, showed that Holladay 1 achieved the lowest MAE (0.346), ahead of T2 (0.348) and SRK/T (0.364). However, the small sample size is a significant limitation of that study [36].

The first evaluation of the trueness of AI-based IOL power calculation formulas (Hill-RBF, Ladas, and FullMonte) in medium-long eyes was provided by Kane et al. in 2017 [16]. That study comprising 340 such eyes showed that Hill-RBF outperformed other AI-based formulas, similar to our outcomes, with that our results are more accurate (MAE of 0.271 versus 0.370, and the percentage of eyes with a PE within ±0.5 D of 86.45% versus 75.0%). This difference results from applying various Hill-RBF versions (we used Hill-RBF 3.0, and Kane utilized the original Hill-RBF). It has been proven that Hill-RBF 3.0 is the most exact version of this formula [43]. Another interesting study on this topic was published by Voytsekhivskyy et al. in 2023. They checked the accuracy of 24 IOL power calculation formulas, including six AI-based in medium-long eyes. In their study, Pearl-DGS achieved the highest percentage of patients with a PE within ±0.5 D, ahead of Kane and Hill-RBF 3.0 (94.83, 94.74, and 91.23, respectively). Those results coincide with ours, except that the order was various (Hill-RBF 3.0, LSF AI, and Pearl-DGS (86.45, 85.51, and 85.05, respectively). Considering MedAE to evaluate the trueness of IOL power calculation formulas, which is consistent with Hoffer’s recommendations [38], they achieved the following outcomes: Hill-RBF 3.0 (0.156), followed by Kane (0.176) and Karmona (0.183). In our study, the lowest MedAE was gained by Hill-RBF 3.0 (0.200), second-lowest by LSF AI (0.210), and third-lowest by Kane (0.228); therefore, they differed only slightly. Variations may result from the type of IOLs, i.e., Tecnis 1 ZCB00 (Johnson & Johnson Vision, New Brunswick, NJ, USA) contrary to Acrysof IQ SN60WF (Alcon Laboratories, Fortworth, TX, USA). However, they tested only 58 such eyes, which is insufficient to carry out a full-fledged statistical analysis.

The only peer-reviewed study on IOL power calculation in medium-long eyes with RMSAE as a main parameter to evaluate the accuracy of formulas was published in 2024 by Stopyra et al. [25]. They examined 20 formulas, of which five were AI-based. In that study, LSF AI achieved the lowest RMSAE (0.270), Hoffer QST the second-lowest (0.298), and Karmona the third-lowest (0.313). In this study, we obtained the order as follows: Hill-RBF 3.0 (0.368), Pearl DGS (0.374), and Hoffer QST (0.378); thus, the outcomes differ significantly. In turn, the results of both studies are similar in terms of the percentage of patients with a PE within ±0.5 D, i.e., LSF AI ahead of Hoffer QST and Karmona (92.74, 90.32, and 90.32, respectively) versus Hill-RBF 3.0 followed by LSF AI and Hoffer QST (86.45, 85.51, and 85.05, respectively). The lack of the Hill-RBF 3.0 formula is the main limitation of that study, so comparing the two is difficult. The same authors, using a similar methodology, compared the accuracy of seven AI-based formulas; however, the study concerned extremely long eyes [50].

We recognize several limitations of our study. First, contrary to Hoffer et al.’s recommendations, both eyes of some patients were examined [51]. However, this should not influence the outcomes since a minimum sample size of 138 eyes to achieve a confidence level of 95% has been provided by various patients. Second, such parameters as ACD, K, and LT were not considered. However, there are reports about essential biases in the PEs of most of the formulas when plotted versus not only AL but also ACD, K, and LT [32]. Third, only one model of IOL was applied in all patients, so these outcomes may not be generalizable to IOL models of various designs. The discrepancies in IOL shape could affect PE and the accuracy of the formula. However, according to Hoffer, using only one IOL model to evaluate the trueness of the IOL power calculation formulas is as good a method as using various lens models accordingly [45]. Fourth, the age of the patient was not studied. It was proved that IOL power calculation formulas, including AI-based ones, may have variable exactness for various age groups [52]. Fifth, not all AI-based formulas were examined. However, the FullMonte method is currently unavailable, Zhu-Lu is only intended for long eyes (AL ≥ 26.0 mm), and the Zeiss AI calculator is available only in the United States. Finally, the pupil dilatation was not examined. Some authors showed the influence of pupil dilation on the trueness of IOL power calculation formulas; however, they do not address AI-based formulas [53].

## 5. Conclusions

This study shows that the Hill-RBF 3.0 formula provides highly accurate results in medium-long eyes. The formula is readily available via its online calculator or on the web page of the European Society of Cataract and Refractive Surgeons. In our series, all seven tested AI-based formulas achieved in more than 80% of eyes a PE within ±0.50 D, which is consistent with the European Registry of Quality Outcomes [2].

## Figures and Tables

**Table 1:** The use of AI in IOL power calculation.

AI Hybrid Formulas	Pure AI Formulas	Universal AI Algorithms
FullMonte Hoffer QST Kane LSF AI PEARL-DGS Zeiss AI Zhu-Lu	Hill-RBF 3.0 Karmona Nallasamy	PLUS method Sramka approach XGBoost Calculator

**Table 2:** Demographics of study subjects.

Demographics	Mean (±SD)	Range
Age	71.88 ± 9.60	43–94
Gender M/F, %	90/124	42.05%/57.95%
Axial Length (mm)	25.17 ± 0.42	24.50–25.97
Corneal Power (D)	42.89 ± 1.42	38.53–48.49
Corneal Astigmatism Magnitude (D)	0.69 ± 0.49	0.00–1.68
Anterior Chamber Depth (mm)	3.44 ± 0.40	2.38–4.48
Lens Thickness (mm)	4.23 ± 0.36	3.14–5.24
Corneal Diameter (mm)	12.26 ± 0.40	11.00–13.10
Central Corneal Thickness (mm)	0.557 ± 0.033	0.442–0.640
IOL Power (D)	17.42 ± 1.98	11.5–22.0

IOL = intraocular lens; SD = standard deviation of the error; D = diopter.

**Table 3:** The RMSAE statistical comparison of the seven formulas for the medium-long (24.50–25.99 mm) eyes applying the Bootstrap-t method with the Holm correction.

Formulas *p* Value	Hill-RBF 3.0	Hoffer QST	Kane	Karmona	LSF AI	Nallasamy	Pearl-DGS
Bootstrap-t method							
Hill-RBF 3.0	------						
Hoffer QST	0.987	------					
Kane	0.948	0.987	------				
Karmona	0.021 *	0.284	0.923	------			
LSF AI	0.987	0.987	0.987	0.686	------		
Nallasamy	0.987	0.987	0.987	0.336	0.987	------	
Pearl-DGS	0.987	0.987	0.826	0.140	0.987	0.987	------

RMSAE = root-mean-square absolute error; *p*-values = calculated probability. α = 0.05 − significance level. * Statistically significant difference with other formulas.

**Table 4:** Refractive outcomes obtained by each formula in medium-long eyes (the mean PE, RMSAE, MAE, MedAE, SD of errors, MAD, optimized constants, and percentage of eyes with PEs within ±0.25 D, ±0.50 D, ±0.75 D, ±1.00 D, and ±2.00 D) for each of the seven formulas.

IOL	Alcon IQ SN60WF											
Axial Length	24.50–25.99 mm											
n	214											
Formula	Optimized Constants (ULIB)	PE	RMSAE	SD	MAD	MedAE	MAE	Eyes within PE (%)				
	Alcon IQ SN60WF							PE ≤0.25 D	PE ≤0.50 D	PE ≤0.75 D	PE ≤1.00 D	PE ≤2.00 D
Hill-RBF 3.0	119.00	0.008	0.368	0.367	0.271	0.200	0.271	61.21	86.45	94.86	98.13	100.00
Hoffer QST	119.00	0.014	0.378	0.378	0.287	0.241	0.288	54.67	85.05	95.79	98.60	100.00
Kane	119.00	0.029	0.387	0.385	0.287	0.228	0.289	55.14	83.64	94.39	98.60	100.00
Karmona	119.00	0.185	0.418	0.374	0.281	0.253	0.318	50.93	81.31	93.46	96.73	100.00
LSF AI	119.00	0.008	0.379	0.378	0.275	0.210	0.275	60.75	85.51	95.33	98.13	100.00
Nallasamy	119.00	−0.021	0.381	0.380	0.286	0.235	0.288	57.01	84.58	93.93	98.13	100.00
Pearl-DGS	119.00	0.038	0.374	0.372	0.280	0.234	0.283	54.21	85.05	95.79	98.13	100.00

IOL = intraocular lens. LSF AI = Ladas Super Formula artificial intelligence. PE = mean prediction error. RMSAE = root-mean-square absolute error. SD = standard deviation. MAD = mean absolute deviation. MedAE = median absolute error. MAE = mean absolute error. D = diopter. n = number of cases.

## Data Availability

Anonymized raw data will be made available to qualified investigators upon written request to the corresponding author.
